# Practical Observations on the Treatment of Acute Diseases; Particularly Those of the West Indies

**Published:** 1797

**Authors:** William Wright

**Affiliations:** Fellow of the Royal College of Physicians of Edinburgh, and of the Royal Societies of London and Edinburgh; and Physician to the Forces in the West Indies.


### MEDICAL FACTS

AND

### OBSERVATIONS.

I. *Practical Observations on the Treatment of acute Diseases; particularly those of the West Indies. By* William Wright, *M.D. Fellow of the Royal College of Physicians of Edinburgh, and of the Royal Societies of London and Edinburgh; and Physician to the Forces in the West Indies. Communicated in a Letter to* Maxwell Garthshore, *M.D. F.R.S. Physician in London; and by him to Dr.* Simmons.

*To Dr.* Garthshore.

Dear Sir,

In compliance with your request, I now communicate to you some observations on the treatment of acute diseases; particularly those of the West Indies.

 I shall

I ſhall begin with the *Typhus*, nervous, ſhip, or jail fever, as it is differently ſtiled by different writers.

In a former letter I remarked to you, that the application of cold water externally had been, for ſome time, practiſed by Dr. Gregory, Profeſſor of Phyſic in this Univerſity, in caſes of Typhus, with remarkably good effect; but he has never carried it to the extent I did in my own caſe, and in that of others, ſeveral years ago *. Inſtead of daſhing cold water on the naked body, as I did, Dr. Gregory orders the bodies of his patients to be waſhed with a ſponge, dipped in cold water and vinegar, at leaſt twice a day. This operation I ſhall call the *Lavatio frigida*. The earlier this mode is practiſed the better; becauſe, in Typhus the patient grows daily worſe; for in the ſecond week there is a great increaſe of fever, and a proportionate loſs of ſtrength: but even then Dr. Gregory has found the application of the wet ſponge act as a charm; nor have *delirium* or *petechiæ* been conſidered by him as any bar to the adoption of this remedy; on the contrary, where theſe have been preſent, and the

* See London Medical Journal, Vol. VII. p. 109.

pulſe pulſe much quickened, he has, by the *Lavatio frigida*, ſpeedily reduced the pulſations from 110 to 90 in a minute, and the delirium and other threatening ſymptoms have ſoon after diſappeared.

About a fortnight ago, a ſtudent of phyſic, who had been ill for ſome days before Dr. Gregory was applied to, had, beſides a great degree of fever and delirium, numerous ſpots, or petechiæ, on his breaſt, belly, and extremities. The *Lavatio frigida* was uſed on the day the Doctor firſt viſited him, and by next morning the delirium had ceaſed, and the petechiæ diſappeared. The pulſe, which on the preceding day had been at 110, was now at 80; and by continuing the application of the wet ſponge now and then, the pulſe became natural on the fourth day after the Doctor firſt ſaw him. Many ſimilar caſes might be adduced from the books of the clinical ward of the royal infirmary.

Succeſsful as this method has been in the hands of Dr. Gregory, and ſome others, beſides mine, I am well aware that much caution and judgment are neceſſary in putting it in practice. In all caſes where there are viſceral obſtructions, cold bathing does much miſchief; and in fevers of this ſort, with inflammatory diatheſis, there is reaſon to ſuſpect topical inflammation of the viſcera; in this laſt caſe, if cold bathing were made uſe of, the patient would run the riſk of his life, and the phyſician juſtly loſe his character. Other methods of treatment muſt therefore be had recourſe to, and theſe I will endeavour to point out, from a ſucceſsful practice in the Weſt Indies, as well as in this country.

In fevers, where there are but ſlight ſigns of inflammatory diatheſis, mild antimonials, as James's powder, the antimonial powder of the ſhops, or antimonial wine, in ſmall and repeated doſes, with occaſional opiates, are generally ſufficient to open the pores of the ſkin, and occaſion a gentle perſpiration. But where theſe or the like mild means are of no avail, there is every probability to ſuppoſe, that topical inflammation, internally, has taken place.

In caſes of this ſort I have immediate recourſe to calomel, either by itſelf, or joined with antimonials or opiates. The quantity of calomel I employ is proportioned to the violence of the diſorder, and the danger the patient is in. In this country I have ſeldom exceeded five or ſix grains of calomel a day; but in the Weſt Indies I have given twenty grains in twenty-four hours with the moſt marked ſucceſs.

In

In 1771, Dr. Lyſons publiſhed his Eſſay on the good Effects of Camphire and Calomel in continual Fevers. In ſuch caſes I have found no occaſion for the firſt of theſe; and Dr. Lyſon's ſucceſs with the latter muſt have been in caſes where there was a morbid and topical affection of the viſcera and alimentary canal.

About fourteen years ago, I communicated my method of treating obſtinate and acute diſeaſes, in the Weſt Indies, to an eminent phyſician who had the care of a large hoſpital in England. He gave calomel, in large and frequent doſes, in fevers that reſiſted the common methods of cure, and found it to anſwer far beyond his expectations. It ſometimes had no other effect than occaſioning a copious ſtool at times; but for the moſt part it acted as a mild diaphoretic and ſedative: a criſis, or favourable turn of the fever, was ſoon brought about, and the patients ſpeedily recovered.

It ſeems hardly neceſſary to mention to you, that in all caſes of Typhus there can be but little hopes of ſucceſs, unleſs the patients are brought into ſpacious and well-aired chambers, and are lightly covered with bed clothes.

In the firſt ſtage of Typhus, briſk ſmall beer may be given plentifully for common drink, or water ſlightly impregnated with vitriolic acid. The ſtrength of the patients ſhould be ſupported by giving them frequently panada, or gruel, with wine. Attention, too, muſt be paid to the ſtate of the belly, and of the other emunctories.

Some late authors, who have written on Weſt-India diſeaſes, have roundly aſſerted, that in tropical countries fevers are not contagious; but whoever has had the care of crowded hoſpitals, of jails, of ſhips of war, or of tranſports full of troops, muſt have ſeen numerous and fatal inſtances of contagion in the Weſt Indies; more eſpecially where cleanlineſs and free ventilation have been neglected.

From cauſes of this ſort a moſt fatal and deſtructive diſorder broke out in the Weſt Indies in 1793, and ſoon after in Philadelphia, viz. the yellow fever. Dr. Ruſh has claſſed this diſorder with remittents; but every one who has practiſed in the Weſt Indies, knows for certain, that the remitting fevers of warm countries are not contagious. From Dr. Ruſh's book, and from the numerous letters of my correſpondents, there remains not a doubt, in my mind, of the yellow fever being Typhus, exalted to a great degree of virulence from climate, mate, ſituation, and other adventitious circum-ſtances.

The yellow fever has appeared in America at different periods, as we learn from Dr. Lining's paper in the Edinburgh Eſſays, Phyſical and Literary, Vol. II.; and it was this ſame diſorder that committed ſuch havock amongſt the troops, under Admiral Vernon, in 1741.

The commencement of this fever, in Grenada, is dated from May, 1793, ſoon after the arrival of a Guinea ſhip from Sierra Leone, the crew of which had been ſo ſickly, that moſt of the ſailors died of the yellow fever, either in the voyage, or ſoon after the arrival of the ſhip. It ſuddenly ſpread over the other Leeward iſlands, and from thence was carried to Philadelphia, Hiſpaniola, and Jamaica.

The firſt account I received of this fever, was from Dr. James Clark, a phyſician of eminence in Dominica; his letter to me is dated July 23, 1793, and runs as follows: "I have been ha-

" raſſed night and day, for a month paſt, by

" attendance on people ill of the yellow fever.

" Since its appearance in this iſland, it has al-

" ready carried off more than a hundred ſail-

" ors, new comers, and emigrants. In its

" progreſs it has been, and ſtill is, as quick

"and fatal as the plague; it often finiſhes its "courſe in forty-eight hours; but if the ſick "get paſt the fifth day, they generally recover."

All the letters I have had from my medical friends agree, that this fever is highly contagious, and that new comers are moſt ſubject to receive it; particularly ſuch as are young, or are addicted to drinking ſpirituous liquors. Next to theſe are the nurſes and attendants on the ſick, who breathe the air in their chambers, or handle their bodies or bedclothes. But ſuch as avoid infected houſes, or keep at a diſtance from people convaleſcent, are no way ſubject to the yellow fever. It appears, alſo, that people of colour, and negroes, are in a manner totally exempted from this diſeaſe, except ſuch as are employed as houſe ſervants, and fare the ſame as white people.

The Creole white inhabitants, and others who have long reſided in the iſlands, are, it ſeems, ſeldom attacked with this diſorder, unleſs under the circumſtances above mentioned. But why the yellow fever ſhould attack ſome, and not others, can only be accounted for in this way: that in order to receive or reſiſt contagion, men's bodies and minds muſt be in a particular ſtate; and that field negroes ſhould not be liable to it is is to me inexplicable. They, however, have their epidemics, from which white people are exempted.

This diſorder ſeems to exert its direful effects on the ſtomach, inteſtines, and other viſcera in general, but particularly on the liver and gall bladder. Sometimes the lungs are greatly affected; and extravaſations have been found in the brain after death.

It is not my intention to delineate the progreſs and ſymptoms of this fever; it will be ſufficient to ſay, that bilious vomitings are amongſt the concomitant and diſtreſſing ſymptoms of yellow fever; and that what is called the black vomiting generally happens towards the fatal termination of the diſeaſe.

I haſten to the medical treatment as practiſed by Dr. James Clark, and others of my friends, in the Weſt Indies. Dr. Clark, in his letters to me on this ſubject, regrets his being called ſo late to the ſick in this fever, twenty-four hours having often elapſed before he has ſeen them: but even at this late period, ſays he, "I have "been lucky enough to ſave three out of four, "or four out of five, of thoſe who had the yel-"low fever." In caſes where he has been called in on the firſt day of the fever, he aſſures me he has ſeldom loſt any one. He firſt endeavours to purge briſkly with ten grains of jalap and ten grains of calomel every three hours. If the vomiting continues, ten grains of calomel, by itſelf, are given, till ſtools are procured; and after this calomel, in doſes of five grains, with or without opium, every third or fourth hour. In urgent caſes he has recourſe to mercurial friction, till the violence of the ſymptoms has abated. "If," ſays he, "I can by "any means introduce a ſufficient quantity of "mercury into the habit in time, ſo as to affect "the mouth and gums, I have no heſitation in "declaring that my patient is out of danger."

Dr. Clark has given ſixty or eighty grains of calomel in three days; Dr. Drummond, a learned and eminent phyſician in Jamaica, has given two hundred grains in the ſame ſpace of time, beſides friction, with ſtrong mercurial ointment, with ſucceſs.

With regard to bleeding, Dr. Clark tells me he has now and then had occaſion to order it in full habits; he has recourſe to this, however, but ſeldom, and then very ſparingly. In Jamaica the lancet is now laid aſide in the treatment of this diſeaſe; as ſome young men, who were ſeized with the yellow fever, and blooded on the the day of the attack, died in a few hours after. The American practice, therefore, will not ſucceed in the Weſt Indies.

In caſes where the ſtrength of the patient is much reduced, the ſtrongeſt wines, or even brandy itſelf, muſt be freely uſed. Dr. Drummond tells me, that in ſuch dangerous ſtages of the diſeaſe, even when the black vomiting has come on, he has given the pepper medicine * with ſucceſs. The uſe of this medicine is continued till a generous warmth takes place, which muſt be kept up ſo long as the delity or the vomiting laſt; but, in the mean time, the uſe of mercury muſt be puſhed vigorouſly, till the mouth is affected, and till there are evident appearances of a reſolution of the diſorder, and an abatement of the moſt violent ſymptoms.

In ſuch ſtages of Typhus, where there were petechiæ, a difficulty of ſwallowing, or a ſenſe of choaking; or where aphthæ were preſent,

* This is compoſed of three grains of powder of Cayenne pepper, made into a pill with mucilage, and may be given every two or three hours; but unleſs the pill is well coated with dough, or white wafer, it will be difficult to perſuade the patient to ſwallow a ſecond doſe.

or or there was a great irregularity of pulse, I have found the use of æther * very beneficial.

Hitherto, the black vomiting has usually been considered as a fatal symptom; and a remedy to obviate it has long been a desideratum amongst physicians. To whom the happy discovery of such a remedy, in the capsicum, is owing I have not yet learned; but he merits the thanks of his country, and of mankind!

That a medicine of so hot and fiery a nature, as Cayenne pepper, can be given with safety and efficacy in a disorder so evidently inflammatory, is truly surprising, and can only be accounted for in two ways: first, by supposing that the stimulus of the pepper is stronger than that of the contagion; or secondly, (to use the language of my late ingenious friend, Mr. John Hunter) that it induces a different action in the stomach and first passages.

On the treatment of intermittents I have but little or nothing new to offer: in such cases I

* For an account of the efficacy of the spiritus vitrioli dulcis in fevers, see a valuable paper, by Dr. Smyth, in the Medical Communications, Vol. I.

have have found every advantage from following the advice of my late excellent friend, Dr. James Lind, of Haſlar, by giving a large doſe of laudanum in the hot fit: this has ſeldom failed to produce a plentiful and kindly diaphoreſis, and the diſorder, in general, has afterwards been cured by the Peruvian bark.

Where intermittents have either been neglected or improperly treated, or where the bark, ſo far from being of ſervice, has ſerved only to load the ſtomach, or has been rejected, I have ſuſpected that ſome viſceral obſtructions exiſted. In ſuch caſes calomel, in ſmall doſes, has had the happieſt effect, and the patients have generally recovered without any other medicine.

Quartans and double tertians, as well as ſimple intermittents, are occaſioned by marſh miaſmata. In warm countries they are frequent, and difficult of cure; and unleſs the ſick are removed to better air, the diſorder will baffle the ſkill of the moſt experienced phyſician. Fevers of this ſort, if even continued but for a ſhort time, occaſion obſtructions of the liver and meſenteric glands, which are too often followed by jaundice, dropſy, and death.

In ſuch caſes, after clearing the ſtomach and primæ viæ, I order mild antimonials, opiates, and and calomel; by theſe means the diſorder is ſoon removed, as I have experienced in a great number of caſes attended with the moſt unfavorable appearances.

The common remitting fevers of tropical countries generally yield to the methods preſcribed by Doctors Cleghorn and Lind, viz. cleanſing the primæ viæ, then giving the bark, wine, and nouriſhing diet; but if they are attended with bilious vomiting, and ſymptoms of inflammatory diatheſis, calomel, in ſmall doſes, (as two grains every three hours) appeaſes the vomiting, opens the belly, and brings on a gentle moiſture on the ſkin. After this the bark may be tried, but I have often ſeen the ſick recover ſooner without it.

Where fiery eruptions, with ſwelling and inflammation, break out in the mouth and lips, at the decline of bilious remittents, quartans, and other obſtinate fevers, Dr. Kirkland juſtly remarks, that the whole alimentary canal is affected with this ſort of eryſipelas. To that author I am indebted for the treatment of the patient in this critical and dangerous ſtage of the diſeaſe. Calomel, either by itſelf, or joined with with mild antimonials and opiates, in ſmall doſes, does every thing that can be wiſhed for. If the eruption has continued any length of time, and degenerated into little ill-diſpoſed ulcers and ſcabs, the *unguentum hydrargyri nitrati* effectually cures them in three or four days.

Remitting fevers, ariſing from marſh miaſmata, are frequently obſtinate and fatal. In many caſes of this kind the tongue is furred and ſlimy, and the vomiting inceſſant, with great head-ach and proſtration of ſtrength. Sometimes I have ſettled the ſtomach with a decoction of camomile flowers; at other times by ſaline draughts, taken in an efferveſcing ſtate; but the moſt effectual remedy I have ever tried, has been a ſlight infuſion of the *Quaſſia Polygama*, or Bitter-wood *; after which the Peruvian bark, or Jeſuit's bark of Jamaica †, has completed the cure.

In bilious remittents I have ſeen a yellow ſuffuſion over the whole body occur in the courſe of the diſeaſe; ſometimes in the firſt ſtage, but

* See the account of this tree by Mr. Lindſay, in the Tranſactions of the Royal Society of Edinburgh, Vol. III. and Medical Facts and Obſ. Vol. V.

† Philoſ. Tranſ. Vol. LXVII.

more frequently towards the end of the fever, which too often terminated fatally.

In 1785, a gentleman* at Hampden eftate, in Jamaica, was feized with a bilious remittent, attended with conftant retching, and vomiting of bile. I was called to his affiftance on the fourth day of his diforder; his fkin was then of a deep yellow colour, and his urine tinged linen cloth, as in jaundice: the whole of his fymptoms indicated extreme danger. My firft object was to procure ftools by means of ftimulating injections, and fmall dofes of the compound powder of jalap; but as the vomiting continued, and the fever remained high, I determined to give him two grains of calomel every two hours. On the following day he was better, but the ufe of the calomel was continued till the evening, at which time his ftomach was fettled. He had two copious evacuations by ftool; the fever was greatly abated, and there was a gentle moifture on the fkin, which I encouraged by fmall dofes of antimonial wine, and watery tepid drinks. After this he recovered daily, but the yellownefs of the fkin continued fome weeks before it wore completely off.

* Mr. Alex. Thoburn, at prefent in Scotland.

There

There are other acute diſeaſes, in warm countries, that are very deſtructive in their nature; amongſt theſe, is the *Hepatitis*, or inflammation of the liver. It is either acute or chronic. In acute Hepatitis there are ſtrong ſymptoms of phlogiſtic diatheſis; and theſe I endeavour to obviate by a moderate bleeding, gentle laxatives, and diluting drinks. The application of a bliſter over the part affected, is ſometimes uſeful. If the fever and pain continue, I preſcribe ſmall doſes of antimonial powder, or antimonial wine, to bring on a gentle perſpiration; ſhould this, however, be not ſpeedily brought about, I loſe no time in exhibiting mercury internally and externally, till the diſeaſe is conquered: and this I have done with uniform ſucceſs for twenty ſeven years; whereas acute Hepatitis, treated by frequent and copious bleeding, too often terminates in Phthiſis Pulmonalis, or ſome other fatal diſorder.

The chronic Hepatitis is very common in Great Britain, and is often miſtaken for Dyſpepſia. Small doſes of calomel, (as a grain at bedtime every night for a fortnight) are in general ſufficient to remove it.

Pleurifies and acute Peripneumonies are common and fatal difeafes in all tropical countries, efpecially amongft the negroes who live upon eftates in the hilly and mountainous parts of Jamaica.

In the cure of Pleurifies, bloodletting is at firft requifite; but a repetition of it requires much caution. Profufe and repeated evacuations of this fort weaken the fyftem; and I have feen many inftances, where an improper ufe of the lancet in fuch cafes has been fucceeded by general debility, pulmonary confumption, and dropfy. In thefe difeafes, after one, or at moft two moderate bleedings, I direct the belly to be opened by clyfters, or fome gentle laxative; give nitre diffolved in the patient's common drink, and advife a thin fpare diet. A blifter applied to the fide affected generally gives great relief. But if the fever is confiderable, and the pain acute, I order from three to fix grains of antimonial powder every two hours, till a plentiful fweat takes place, which I encourage by a liberal ufe of warm tea, or water gruel. If fmall dofes of the antimonial powder have not the defired effect, I give ten, fifteen, or twenty grains grains for a dofe; nor am I afraid of exciting full vomiting, either in Pleurify or Peripneumony; on the contrary, fuch dofes have proved highly beneficial.

When the diforder has refifted thefe means, I have ordered, with great fuccefs, calomel, in large and frequent dofes, as long as the violent fymptoms continued.

Pleurifies and peripneumonies are often epidemic amongft the negroes in Jamaica, and attended with a remitting fever. Full vomiting is here particularly ufeful; in the exacerbations twenty-five or thirty drops of laudanum take off the fpafm, and the bark fecures the patient from a return of the complaint.

I might have mentioned *fplenitis* and other internal inflammations, but as they give way to fimilar management, I proceed to treat of the dyfentery.

The dyfentery has in every war carried off more of our troops in the Weft Indies, than all the other difeafes of that climate. It is a melancholy truth, that this fatality is greatly owing to the folly and intemperance of foldiers and failors, and not to the climate, which has been blamed for it. Drinking to excefs of new and bad rum, deftroys the powers of the ftomach, and debilitates their ftrength: they are either attacked by fome violent inflammatory diforder, or are liable to receive infection from human bodies, or from marfh miafmata.

Europeans labouring under dyfentery, in the Weft Indies, have more or lefs of remitting fever: in fuch patients bleeding, if at all neceffary, ought to be had recourfe to very fparingly. Negroes ill of dyfentery, or other acute difeafes, admit of a more free ufe of the lancet. In ordinary cafes, an emetic of ipecacuanha, afterwards a dofe of rhubarb and calomel, and an opiate at bed-time, generally carry off the diforder.

In epidemic dyfenteries, attended with great proftration of ftrength, and other fymptoms of putrefcency, I am folicitous to purge off the offending matter in the alimentary canal, and afterwards to correct the difpofition to putrefcency: for this purpofe I prefcribe a ftrong decoction of tamarinds; in two pints of which I order two ounces of purging falt to be diffolved: an ordinary tea-cup full of this is directed to be taken every three or four hours, till it has operated operated plentifully by ftool; after which, at bed-time, I give an opiate. On the following day the decoction of tamarinds, without the falts, is given; or the fick are allowed to eat preferved tamarinds, as they think proper.

In cafes where this method has failed of fuccefs, I have had recourfe to a mixture of vegetable acid and purified fea falt, an account of the preparation and good effects of which I feveral years ago communicated to the American Philofophical Society, who have inferted it in their Tranfactions *: it is compofed of lemon or lime juice three ounces; of fea falt purified an ounce, or as much as the acid will diffolve; of any fimple diftilled cordial water one pint; and of loaf fugar a fufficient quantity to fweeten it: of this a wine-glafs full may be given to adults every two, four, or fix hours.

A moft refpectable author defines dyfentery to be a fever of the inteftines, and for the cure of it prefcribes antimonials and opiates, which in flight cafes I have known to anfwer. This idea of the difeafe comes very near to my own;

* See Tranfactions of the American Philofophical Society, Vol. II. 4to. Philadelphia, 1786; and London Medical Journal, Vol. VIII. p. 97. 8vo. London, 1787.

but when dyſentery is attended with phlogiſtic diatheſis, the fever is rather the *effect* than the *cauſe* of the diſorder. Diſſections of ſuch as have died of dyſentery, have evidently ſhown, that inflammation, and conſequent gangrene, had taken place in the ſmaller inteſtines, as well as in the colon.

In dyſenteries where the fever has been conſiderable, the tongue dry and parched, the gripes ſevere, and the ſtools very frequent, with ſcarcely any thing elſe than blood or mucus, I have preſcribed, with good effect, calomel, in doſes of five grains, every ſix hours, till a copious ſtool or two has been procured; and afterwards in ſmaller doſes, with occaſional opiates, while the fever and gripes have continued.

Autumnal dyſenteries in this country have generally given way to ſome one or other of the correctors I have mentioned above; but particularly to an infuſion of Quaſſia Polygama *, or Bitter-wood; after which I have preſcribed the Peruvian bark to ſtrengthen the ſyſtem.

* There is no ſuch thing in the ſhops as Quaſſia Amara; it is the Bitter-wood or Bitter-aſh that is imported, and anſwers every purpoſe, perhaps better than the Quaſſia Amara.—Vide Medical Facts and Obſ. Vol. V.

In the treatment of the different diſeaſes mentioned in this paper, you have ſeen the liberal uſe I make of calomel. I have contented myſelf with candidly relating to you the effects I have experienced from it, without attempting any theory of the mode in which theſe effects are produced. I think it neceſſary, however, to obſerve to you, that freely as I have adminiſtered calomel in different acute diſeaſes, I have ſeldom, if ever, been ſurpriſed with a ſudden ſalivation. I indeed have paid daily attention to the ſtate of the mouth and gums, and as ſoon as I have obſerved the latter ſpongy, and that the tongue was beginning to be moiſt about the edges, I have deſiſted from the farther uſe of calomel; becauſe I was then certain that a reſolution of the diſorder was begun, and that my patient was out of danger.

In anſwer to your queſtion, how early I got the firſt hint of the uſe of calomel in fevers? I anſwer, it was my good fortune, for many years, to enjoy the friendſhip and confidence of the late Dr. Lind, of Haſlar; and it was from his converſation, and the information contained in his excellent work on the diſeaſes of warm climates, that I learnt the Eaſt-India practice of giving mercury in inflammations of the liver, and the ſucceſs with which the late Sir John Eliot had treated viſceral obſtructions by the ſame remedy; all which I knew ſo early as the year 1760. But it was not before 1764 that I began to give calomel in ſo free a manner as I have done ever ſince, not only in hepatitis, but in all the other acute diſeaſes I have treated of; and I extended its uſe from reaſoning in my own mind, and from analogy. I have never had cauſe to repent of the further trials I made; but, on the contrary, have had reaſon to conſider this practice as the happy means of ſaving the lives of a great number of people.

I think it right to add, that Dr. Drummond, of Weſtmoreland, in Jamaica, whom I have already had occaſion to mention more than once in the courſe of this letter, began to adminiſter calomel in fevers and pleuriſies as early as I did, though without our having had any communication on the ſubject with each other. I have ſince found that he learned the uſe of it, in ſuch caſes, from Dr. Smith, a phyſician at Savannah le Mar, who was in the habit of giving it, in doſes of twenty grains, in acute diſeaſes, with great ſucceſs.

These observations are extended to a greater length than I at first intended. After all, you must consider the whole only as hints for the treatment of acute diseases, and if you are of opinion that they will be useful, you have my consent to make them public.

I have the honour to be,

With the greatest esteem and regard,

Dear Sir,

Your most obedient servant,

WILLIAM WRIGHT.

*Edinburgh,*

*December* 10, 1794.

II. *Facts*

